# Overnutrition is a significant component of food waste and has a large environmental impact

**DOI:** 10.1038/s41598-022-11813-5

**Published:** 2022-05-17

**Authors:** Silvio Franco, Marco Barbanera, Roberto Moscetti, Clara Cicatiello, Luca Secondi, Riccardo Massantini

**Affiliations:** 1 Department of Economics, Engineering, Society and Business Organization, University of Tuscia, via del Paradiso 47, Viterbo, Italy; 2 Department of Innovation in Biological, Agro-Food and Forest Systems, University of Tuscia, Via San Camillo de Lellis Snc, Viterbo, Italy

**Keywords:** Environmental impact, Climate change

## Abstract

Food waste and obesity and overweight conditions are both linked to the unsustainability of current food systems. This article argues that overnutrition should be considered a form of food waste and it provides a first estimation of the quantity of food over-consumed in Italy. This is done by calculating the excess calories consumed by obese and overweight people and converting them into food quantities by comparison with a typical Italian diet. The total quantity of food consumed in excess by Italian citizens due to overnutrition is calculated as 1.553 million tonnes per year, which is comparable to the current national household food waste assessments. The environmental impact arising from production and consumption of this food accounts for 6.15 Mt of CO_2_-eq per year, as estimated by a Life Cycle Analysis conducted on the 46 food categories which compose the typical Italian diet. Overnutrition in the South-Islands regions of Italy exerts the largest impact (31.6%), followed by the North-West (26.6%), the Centre (22.2%), and the North-East (19.1%).

## Introduction

With the rise in global obesity level, an increasing number of people constantly consume food beyond the recommended caloric intake^1^. The current definition of Food Waste (FW) does not include overnutrition, although several authors support the idea that the food consumed in excess of individual needs should be accounted as a waste^2,3^.

The discussion about overnutrition and FW is gaining attention as both phenomena are key challenges for the sustainability of current food systems. On the one hand, health problems resulting from being overweight or obese now affect more than 2 billion people^4^ and the growing debate about the worldwide obesity epidemic is pushing policy makers and food chain actors to take action. Although obesity is not explicitly featured in any of the Sustainable Development Goals it has been defined “*a Ghost at the Feast*”^5^ because many of the goals are linked to the reduction of obesity. On the other hand, FW is currently estimated as 17% of the food available at the retail and consumer level, accounting for 931 million tonnes per year, 61% of which occur at the consumer level^6^. FW is considered a major challenge for the sustainability of food systems, and it is directly addressed by the Sustainable Development Goal target 12.3, that seeks to halve per capita global FW by 2030. With the food sector representing a major contributor to greenhouse gas (GHG) emissions, about 18 Gt CO_2_ eq. in 2015, representing 34% of total GHG emissions^7^, it is now recognized that the FW issue entails a relevant environmental impact^8^.

There is increasing evidence that a remarkable amount of food is consumed in excess due to the higher energy demands of obese and overweight individuals^9^. The production and consumption of such food puts pressure on natural resources and on the environment^10^, just as the food that is lost and wasted at different stages of the supply chain^11^. It should also be noted that if overnutrition was not considered a component of FW, then eating more food than needed could be considered a strategy to prevent wasting food^12^ and for the pursuance of food security. This would clearly create a huge paradox in a world that is struggling with the obesity epidemic. Despite the growing consensus around this theoretical approach, assessing the magnitude of this “new” component of FW is critical to understanding its relevance and role. This article presents the results of a first quantification of the food wasted due to overnutrition in Italy, and its environmental impact.

## Overweight conditions in Italy

In Italy in 2019, 43.5% of males and 28% of females were overweight, while 11.7% and 9.8% were obese^13^. This figure is underestimated because it only refers to people aged over 18, while we know that overweight conditions are also very common in childhood^14^. The trend of overweight populations is increasing worldwide; a rise in obesity of 11.2% amongst men and 8.6% amongst women was observed between 1991 and 2018, together with an even higher rise in the numbers of overweight people (+ 69.1% among males, + 53.3% females)^15^.

Such conditions are closely linked to overnutrition: that is, excessive consumption of food. The relationship between overweight and overnutrition is bi-directional: on the one hand, overnutrition is a cause of overweight and obesity; on the other hand, overweight/obese people require a greater food intake to maintain their (increased) basal metabolism. Edwards and Roberts^16^ estimate that obese people have an + 18% food consumption with respect to a standard diet. In this article we argue that such overconsumption of food represents a form of FW, which adds up with the food lost in the upstream stages of the supply chain.

With the aim of understanding whether the amount of this form of FW is significant—both in absolute terms and with respect to other forms of consumer FW—we attempted a first estimate based on Italian data of overweight conditions.

## Results

### Extent of overnutrition in Italy

The first result of this study is an estimation of the extent of overnutrition in Italy, based on national data about overweight conditions. Such estimation assumes that overnutrition is the additional caloric intake required to maintain an overweight/obese person in her/his current condition. Overnutrition is thus evaluated as the difference between the calories ingested and the recommended calorie intake^17^ or, in other terms, the energy gap between the food consumed and the food required^11,18^. Such difference was estimated considering the body mass index of the overweight/obese people, broken down by gender and age classes, as well as different levels of physical activity for the different categories of individual (see “Methods” section for more detail).

Overall, we estimated that overnutrition accounts for 2.676 billion kcal per year for the Italian population, 64% of which is due to the increased caloric intake of overweight people. Significant territorial differences can be detected in this estimation with 32% of the total overnutrition occurring in the South of Italy, while North-West, North-East and the Centre of the country account for 25%, 20% and 22% of the total, respectively.

### Quantity of food corresponding to overnutrition

To estimate the quantity of food corresponding to the excess calories consumed by Italian overweight and obese people, we have pieced together the food categories—and the respective quantities—that make up a typical Italian diet.

**Figure 1:**
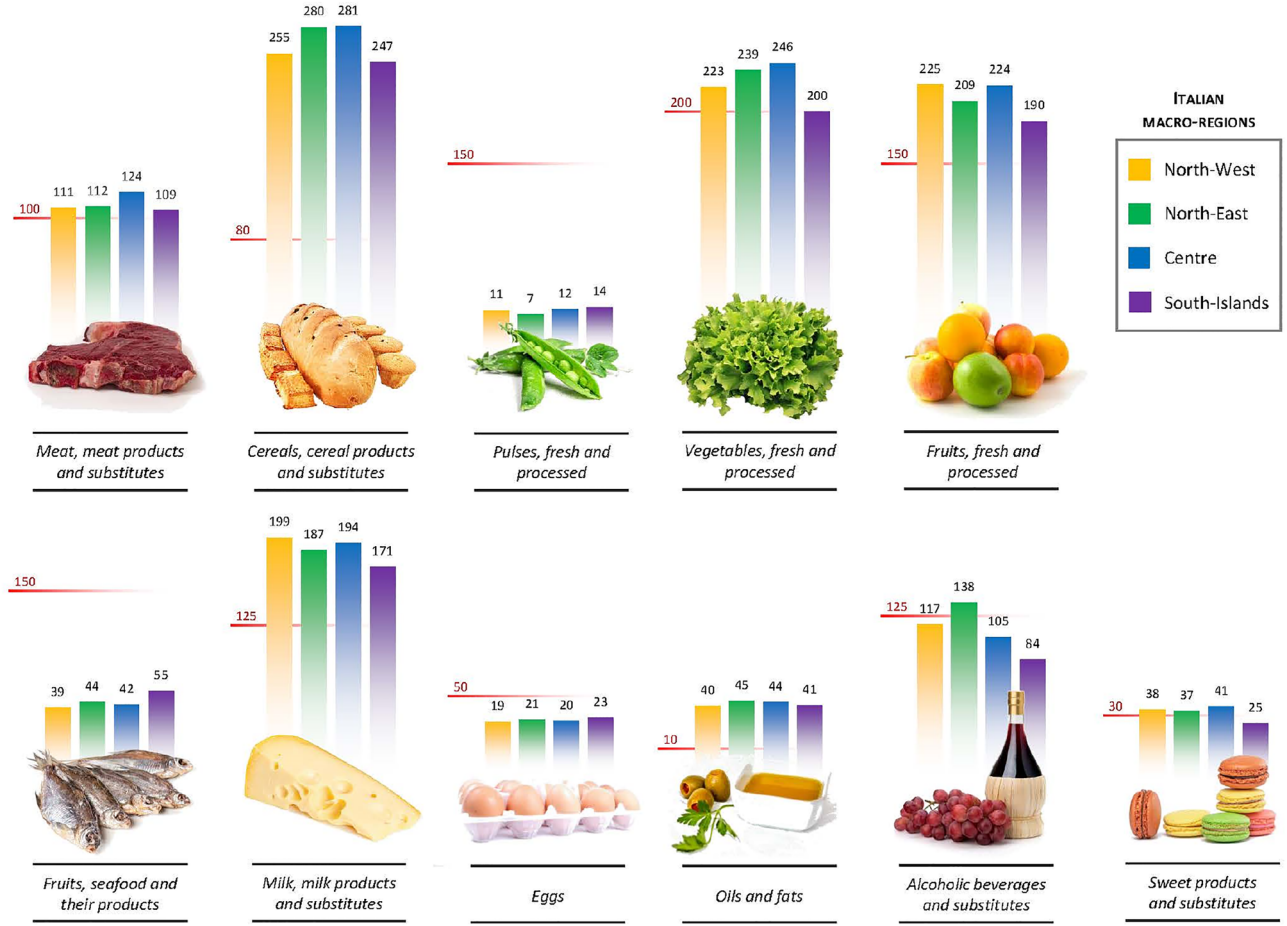
Mean individual daily consumption by food category in male and female adults (18–64 years) in the four Italian macro-regions (INRAN-SCAI, 2005–2006). Numbers are expressed in grams per day. Red horizontal lines represent the standard portion sizes for each food category, expressed in grams per day.

Based on the third Italian National Food Consumption Survey (INRAN-SCAI 2005–2006, more info at www.crea.gov.it), the present study identified the main features of the typical Italian diet in 11 large categories and 46 subcategories of foods and beverages. Figure 1 shows the average daily food consumption by food category of adult males and females (18–64 years) in the four main geographical areas of Italy (North-West, North-East, Centre and South-Islands). The same figure includes examples of standard portion sizes per food category, determined using the fourth edition of the LARN (Level Assumption Recommended Nutrients) for the Italian population^19^. They represent reasonable quantities of food, consistent with food tradition and consumer expectations, taken as reference units for both nutrition professionals and consumers. At first glance, the daily food consumption pattern is characterized by the highest contribution from cereals and cereal products, oils, and fats, followed by meat, vegetables, fruits, alcoholic beverages, and sweets. On the other hand, daily consumption of pulses, fish and eggs is substantially lower than the standard portions of 150 g fresh pulses, 150 g fresh fish and 50 g eggs, respectively. Food consumption data shows similar patterns among the four main macro-regions, which were otherwise characterized by a tendency towards a lower daily food consumption in the South-Islands area with some exceptions (i.e., fish, eggs, oils, and fats). The caloric content of such diets is shown in Table 1, where the specific caloric content of each food included in the diet is used to calculate the total caloric content.

**Table 1:** Caloric content of the typical Italian diet by macro-region. The table reports the weight of the typical diet expressed in grams per day, its caloric content expressed in kcal, and the conversion rate obtained by dividing the weight of the daily diet by its respective caloric content, expressed in grams per kcal.

Macro-region	Weight of the typical daily diet (g)	Caloric content of the typical daily diet (kcal)	Total conversion rate g/kcal
North-West	1216	2067	0.59
North-East	1252	2178	0.57
Centre	1282	2176	0.59
South-Islands	1121	1960	0.57

For each area, a conversion rate, expressed in grams of food for each kcal, is calculated as the ration between the two. This conversion rate supports the conversion of kcal into quantities of food.

**Figure 2:**
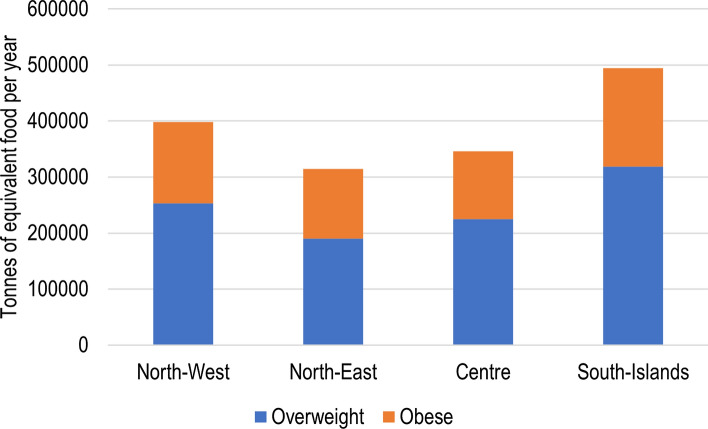
Quantity of equivalent food consumed in excess in Italy, in 1 year, due to overnutrition of overweight and obese people in Italy, by macro-region. Numbers are expressed in tonnes per year, and they refer to the whole population of each region. The total food consumed in excess adds up to 1.553 million tonnes of food in the whole country.

**Figure 3:**
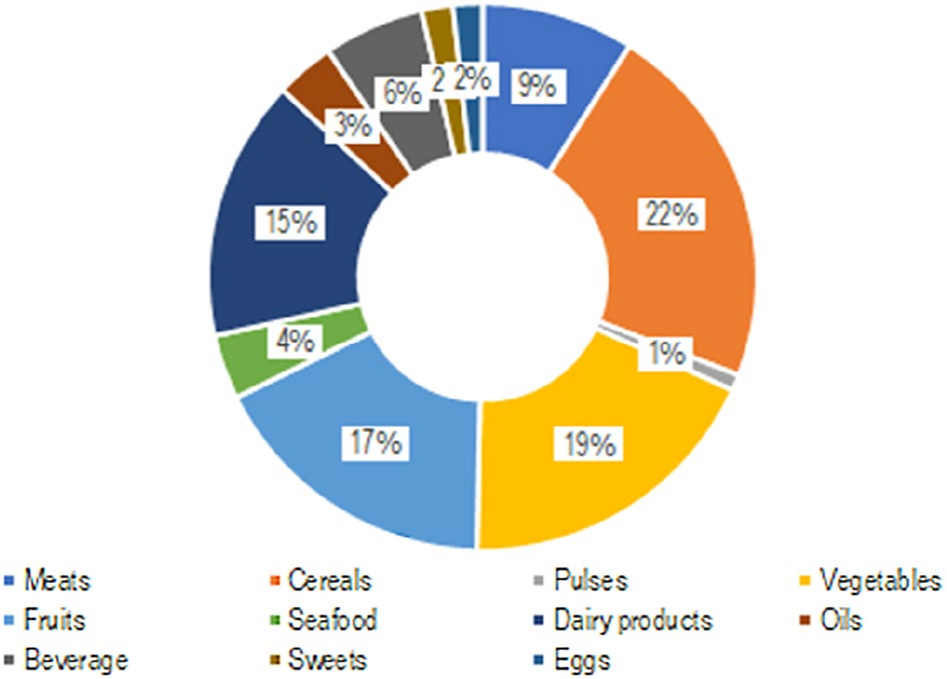
Contribution of food categories to overnutrition of overweight and obese people in Italy. The Figure represents the percentage contribution of each food category to the total food consumed in excess in the whole country.

The conversion rate between caloric content and quantity of food has been used to calculate the equivalent quantity of food corresponding to the excess calories intake consumed by Italian overweight and obese people. Such estimation is made for all the 46 food categories considered in the typical diet, summing up to 1.553 million tonnes of equivalent food over-consumed in Italy every year (Fig. 2). The food categories that are most represented (Fig. 3) are cereals, fruits, and vegetables, which together make up almost half of the total food over-consumed. No significant differences can be detected among the Italian macro-regions concerning the prevalence of such food categories in overnutrition.

### Environmental impact of overnutrition

The GHG emissions associated with overnutrition in Italy are calculated for each Italian macro-region, distinguishing between overweight and obese people, and splitting the environmental impacts among the supply chain stages of each foodstuff. In particular, the following stages are considered: production (including primary agricultural and fishery production), transformation (including food processing), packaging (including production of primary packaging and its end of life), transport (including raw material transport to processing plant or distribution centre and transportation from processing place to distribution centres), distribution (including transportation from distribution centres to retailers and the storage at the distribution centre and at the supermarket) and consumption (including the cooking phase of food). The GHG emissions considered for each stage are reported in the Supplementary Information.

The total GHG emissions of overnutrition in Italy are estimated at 6.15 Mt of CO_2_-eq per year (Table 2), which corresponds to an additional emission burden of about 24% and 12% for obese and overweight adults than normal-weight people, respectively. On average, each normal-weight person’s food-related emissions in 2018 were 1.75 t CO_2_-eq, with differences in the range of − 7.9% and + 5.4% among Italian macro-areas. This data is comparable with that reported by Crippa et al.^7^ who estimated 2.4 t CO_2_-eq in 2015 for the European region (but including GHG emissions associated with land use and land-use change activities). The results are also aligned with the value obtained by Battle-Bayer et al.^20^, equal to 1.6 t CO_2_-eq per capita for an average Spanish adult citizen. However, comparisons among different studies are difficult mainly due to the differences in terms of functional units (mass-based or energy-based) and system boundaries.

**Table 2:** Total GHG emissions of overnutrition in Italy, broken down by food categories, and calculated for each macro-region and for Italy as a whole. All data is expressed in t CO_2_-eq per year.

Food category	North-West	North-East	Centre	South-Islands	Italy
Beef meat	648,929	397,919	511,362	601,646	2,159,856
Beer	6095	5496	4435	9167	25,193
Biscuits	26,056	18,724	20,145	19,726	84,651
Bread	18,792	20,198	21,197	29,651	89,838
Breakfast cereals	1119	858	923	1506	4406
Butter	30,449	27,229	12,555	13,660	83,893
Cheese	119,200	89,843	95,023	163,099	467,165
Chocolate	17,963	9179	9876	16,117	53,135
Citrus fruits	13,138	9324	8026	16,045	46,533
Cocoa	8771	6723	7233	0	22,727
Crackers	6122	5162	4039	6592	21,915
Dessert	1200	920	990	1616	4726
Dried fruit	3432	3507	2830	3079	12,848
Eggs	19,999	16,942	17,360	32,581	86,882
Fresh fish	99,233	85,281	84,314	198,305	467,133
Fresh leafy vegetables	16,347	14,164	15,532	18,174	64,217
Fresh tomatoes	12,487	11,586	13,820	20,343	58,236
Ham	71,851	59,007	59,253	100,154	290,265
Ice-creams	7937	6083	8330	5826	28,176
Legumes	2244	1095	2019	3844	9202
Liqueurs	1497	1434	617	1007	4555
Marmalade	2108	1615	1738	1418	6879
Milk	51,618	36,578	45,378	64,226	197,800
Offal	20,765	7958	17,124	13,973	59,820
Olive oil	18,325	14,952	18,036	27,844	79,157
Other fats	556	853	459	749	2617
Other fresh fruit-bearing vegetables	17,460	14,170	15,245	20,734	67,609
Other fresh vegetables	12,248	8930	10,593	15,278	47,049
Other fruits	29,043	21,050	26,558	33,238	109,889
Other meats	84,045	90,188	69,308	135,733	379,274
Other oils	1333	1532	1649	2691	7205
Other processed vegetables	15,153	12,556	13,509	18,188	59,406
Pasta	54,509	46,044	48,621	85,338	234,512
Pizza	10,260	1311	8461	6904	26,936
Pork meat	30,434	37,907	53,333	87,039	208,713
Poultry meat	40,731	36,729	47,420	67,716	192,596
Preserved fish	10,971	8409	9047	12,656	41,083
Rice	8771	4802	4822	7308	25,703
Root vegetables	8529	6810	6154	6217	27,710
Spices	612	469	505	824	2410
Sugar	6378	4644	5260	6438	22,720
Sweetness	26,003	15,186	19,402	23,331	83,922
Tropical fruits	9869	6051	7324	9297	32,541
Wheat flour	6472	6106	5337	8264	26,179
Wine	15,199	13,070	13,145	14,468	55,882
Yogurt	22,786	16,338	13,941	10,882	63,947
Total	1,637,039	1,204,932	1,362,248	1,942,892	6,147,111

With respect to the contribution of Italian macro-regions to the environmental burden due to overnutrition, it is interesting to highlight that the South-Islands is the macro-region with the largest impact (31.6%), followed by the North-West (26.6%), the Centre (22.2%), and the North-East (19.1%).

**Figure 4:**
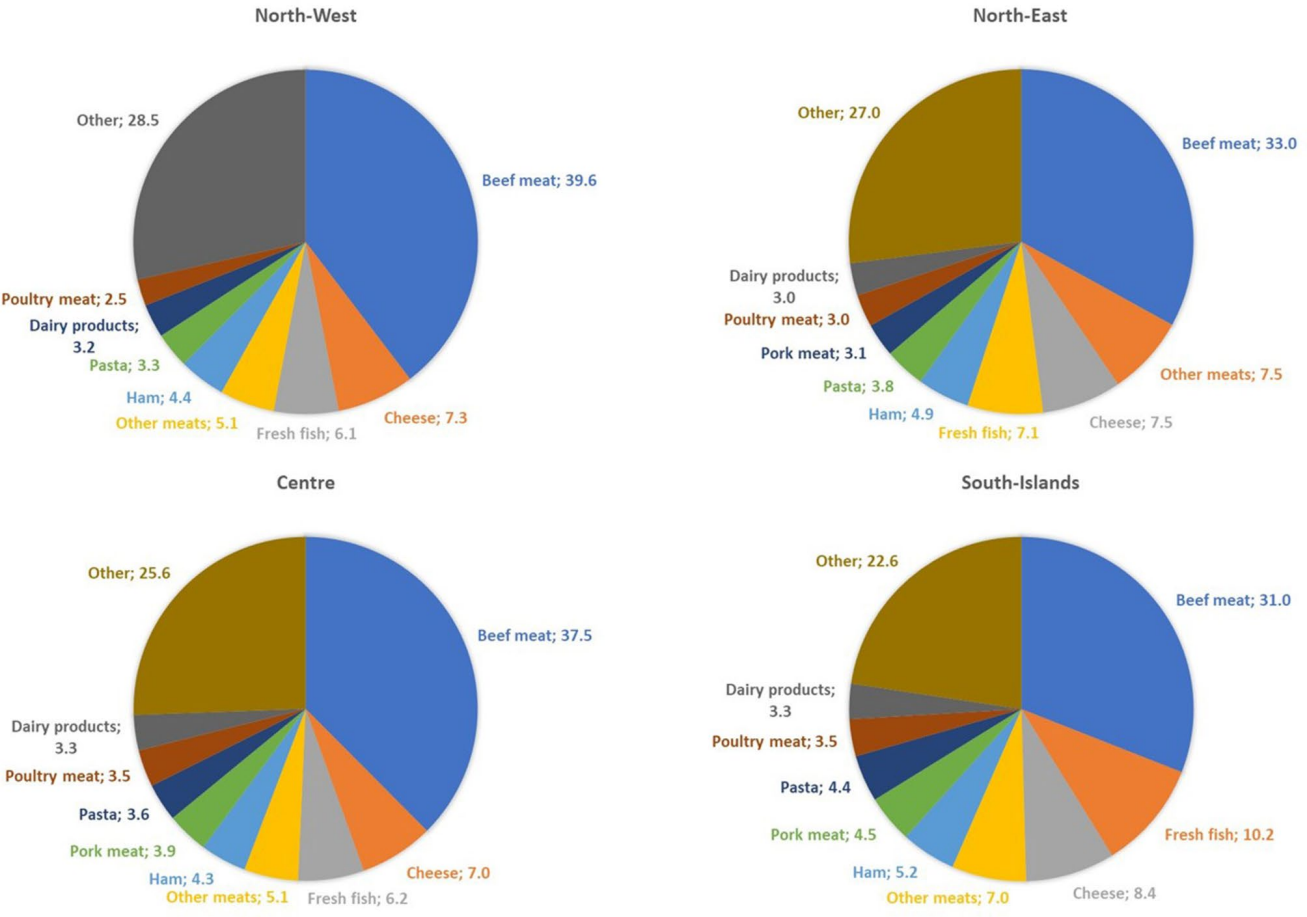
Contribution of food categories to the GHG emissions of Italian macro-regions. The Figure represents the percentage contribution of each food category to the total GHG emissions of the food consumed in excess, in each macro-region of Italy.

Figure 4 shows the contribution of the food categories to GHG emissions due to increased food intake. In all Italian macro-regions, the consumption of beef was responsible for the largest environmental impact of overnutrition, despite accounting for only 3.2% of the food weight at national level. Cheese, fresh fish, and other meats (rabbit and lamb) followed in terms of importance but with different contributions to the total GHG emissions. The most significant differences were related to South-Islands and North-East macro-regions, where fresh fish and other meats were the second-largest contributors, accounting for 10.2% and 7.5%, respectively. Overall, animal-based products were the largest contributors (meat: 55%; dairy products: 13%; and fish: 8%) to the environmental impact of the overnutrition in Italy, followed by fruits and vegetables (9%), cereal-based products (7%) and sweets (5%).

In terms of life cycle processes, the primary production is the phase that has the largest impact on total GHG emissions (82.3%), followed by transportation (6.0%), packaging (4.8%), food processing stage (4.0%), preparation (e.g., cooking, 1.9%), and distribution and retail (1.0%). It should be noted that the farming stage of beef production accounts for about 34% of total GHG emissions associated with Italian overnutrition. In the Agribalyse database, the production of beef was assumed to come 100% from France and the emission factor for the production stage was equal to 43.09 kg CO_2_-eq per kg of (boneless) cooked meat, corresponding to 14.32 kg CO_2_-eq per kg of live weight. To evaluate the reliability of this data for the calculation of GHG emissions due to beef consumption in Italy, the countries of origin and their contribution to the national supply of beef were considered. Basing on data provided by the FAO Food Balance Sheets^21^ and ISMEA^22^, it was determined that most of the beef consumed in Italy in 2018 was produced in Italy (61%), Poland (7%) and France (7%). Buratti et al.^23^ reported an environmental impact of 18.21 kg CO_2_-eq per kg of live weight in Italy, while Bieńkowski et al.^24^ estimated a value of 25.43 kg CO_2_-eq per kg of live weight in Poland, highlighting that the emission factor is acceptable even if slightly underestimated. Considering the emission factor for the beef production in Italy, the GHG emissions associated with the consumption of beef meat would increase from about 2.16 Mt of CO_2_-eq to 2.74 Mt of CO_2_-eq. Moreover, the total GHG emissions of overnutrition in Italy would reach the value of 6.72 Mt of CO_2_-eq, with an increase of about 10%.

## Discussion

Overnutrition is at the same time a factor and a consequence of the obesity epidemic: more and more people consume food in excess, thus incurring in obesity and overweight conditions; at the same time, obese and overweight people need more food than normal weight people to maintain their body. Based on Italian national statistics, the total excess calories consumed due to overnutrition can be calculated and converted into equivalent quantities of food, based on a typical Italian diet. The quantity of food corresponding to overnutrition reaches 1.553 million tonnes, for a total environmental impact of 6.15 Mt of CO_2_-eq every year. Such food can be considered ‘wasted’^2^ especially because, if not, eating more food than needed would be a solution to avoid part of the FW generated at the consumer level. Such a “new” component of the FW stream is very significant: in Italy it almost equates to the FW stream produced at a household level, which is 1.6 million tonnes per year according to Giordano et al.^25^ and represents the main FW stream within the food chain.

The analysis points out that overnutrition has a significant environmental impact in Italy, thus calling for a greater attention to prevent and manage obesity to decrease GHG emissions both on nationally and globally. Just to put some context around these figures, the total GHG emissions in Italy, excluding emissions and removals from land use, land use change and forestry, were equal to 418 Mt of CO_2_-eq in 2019, decreasing by 19.4% from 1990^26^. Therefore, overnutrition accounts for almost 1.5% of the total Italian CO_2_-eq emissions, corresponding to about 21% of the GHG emissions of the agriculture sector. Of course, not all GHG emissions due to overnutrition are produced in Italy because food products are partially imported; this is, however, not a negligible contribution. Furthermore, the average additional GHG emission cost per capita among all macro-regions in Italy was 121 kg/year of CO_2_-eq due to greater food and drink consumption, to which other 476 kg/year of CO_2_-eq per capita could be added due to the increased fuel use to transport the greater body weight of an obese person compared with a person of normal weight^27^.

As this is a first exploratory study on the assessment of overnutrition, some limitations should be considered while interpreting the results. First, the assessment of the calories consumed in excess due to overnutrition is based on a set of assumptions (explained in detail in the methodology) that may change over time along with the people’s lifestyle. Second, the conversion of calories into food quantities is based on a standardised diet that is only relevant for the Italian context. If this study is repeated in other countries, the dietary pattern of the specific context should be used instead. Third, the assessment of the environmental impact of overnutrition performed in this study focused on the climate impacts, but other impact categories may also be relevant and might be considered in similar studies.

## Conclusions

This article argues that overnutrition, that is the consumption of food exceeding an equilibrated calorie intake, must be considered a form of FW. This study provides the first assessment of the extent of this type of FW, showing that, in Italy, it is comparable with the amount of household FW. Even if overnutrition does not generate a disposal of food, it significantly contributes to the overall environmental impact of the food chain, mainly in terms of GHG emissions.

Therefore, overnutrition deserves the same attention as all the other forms of FW, calling for a deeper knowledge of its social, environmental, and economic implications, both at the individual and collective level.

This analysis provides several suggestions to boost the public debate on this topic.

First, it is worth studying overnutrition more in-depth, even beyond the health problems related to overweight and obesity, focusing on its environmental impact and on the implications for food production. Many scholars and policy makers argue that agricultural productivity should be increased to assure food security in the next decades; instead, in countries where the rate of overweight and obese people is higher, this strategy is likely to further feed overnutrition rather than ensuring a more equitable access to food.

Second, a greater awareness of the extent of overnutrition, and its environmental implications, can help spreading and adopting more sustainable food consumption styles. At the individual level, an increased sensitivity towards environmental issues is likely to encourage more attentive behaviours with respect to excessive consumption of food. Even more important, a greater awareness of the consequences of overnutrition at the societal level might help changing the structure of the “obesogenic environment” thus modifying the underlying factors of the obesity problem.

## Methods

### Study design

The estimation of the quantity and environmental impact of overnutrition in Italy followed a three-step approach. The study moved from national statistics about overweight conditions to calculate the kcal consumed in excess, every year, by overweight and obese people, with respect to people with normal weight (step 1). These kcal were then converted into food quantities by assuming that the consumption of food exceeding the individual needs was in line with a typical diet (step 2). Such calculations produced the quantity of each food category that is consumed in excess by overweight and obese people every year, thus providing an estimation of the quantity of food that is wasted because of overnutrition. Such quantities of food were used to develop an analysis of the environmental impact (step 3) that is caused by overnutrition.

All the methods have been performed in accordance with relevant guidelines and regulations.

### Estimation of the quantity of food waste due to overnutrition in Italy

Following Edwards and Roberts^16^, who estimate that obese people have overconsume food by 18% with respect to a standard diet, this study assumes that over-nutrition is the additional caloric intake required to maintain an overweight/obese person in his/her current condition. Then, in this study over-nutrition is not considered as the cause of extra weight, but rather as an effect of it.

Over-nutrition is consequently evaluated as the difference between the calories ingested and the recommended calorie intake^17^ or, in other terms, the energy value of the food consumed and required^11,18^.

To estimate the number of calories over-ingested by overweight/obese Italian people three steps were followed.

1. Estimation of the number of overweight and obese Italian people by gender, age class and macro-region of the country based on data provided by the National Institute of Statistics^13^ (Table 1). To detect overweight and obesity conditions the Body Mass Index (BMI), given by the ratio between weight (kg) and square of height (m), was used. A BMI ranging from 18.5 to 25 is associated with a normal weight, values between 25 and 30 indicate an overweight conditions, and greater than 30 indicates obesity^28^ (Table 3).
2. Evaluation of the average daily energy requirement (ER) by gender, age class and macro-region for normal weight, overweight and obese individuals. The ER is calculated by multiplying basal metabolic rate (BMR) and physical activity level (PAL)^29–31^.
  BMR (kcal/day) is the minimum amount of energy required to maintain life functions^30,32^. To assess BMR the Harris-Benedict equation^33,34^ was used:
  $${\text{Males}}:\quad {\text{BMR }} = { 66}.{473 } + { 13}.{\text{7516 w }} + { 5}.00{\text{33 h }}{-}{ 6}.{\text{775 y,}}$$
  $${\text{Females}}:\quad {\text{BMR }} = { 655}.{1 } + { 9}.{\text{563 w }} + { 1}.{\text{8496 h }}{-}{ 4}.{\text{6756 y}},$$
  where w is the weight (kg), h is the height (cm), and y is the age (years).
  In our study, y is the average value of age classes and h was estimated for each age class starting from data on average height of the Italian male and female population in the last century in each one of the four macro-regions^35^. To estimate w, the inverse formula of BMI (w = BMI × h^2^) was used; the average BMI values (see Table 4) were determined using eight (males/females for each one of the four macro-regions) BMI curves built supposing a normal distribution and maximizing their fit to data on the share of normal weight, overweight and obese people^36^.
  PAL, which expresses a person’s daily physical activity, it is measured through a coefficient that assumes different values for sedentary, active, or vigorous lifestyles^30^. In this study the PAL coefficient was established considering a progressive reduction in the level of activity from younger (moderate/light) to older (sedentary) age groups. The same PAL was associated with different BMI categories, even if it is reasonable to suppose that in general overweight and especially obese people have lower levels of physical activity.
3. A comparison of ER for overweight and obese persons, as calculated in step 2, with the corresponding ER of normal-weight persons was performed to estimate the individual daily caloric surplus for overweight and obese people by gender and age class (see Supplementary Information). These results were used to estimate the total over-nutrition of overweight and obese people (million kcal/year) in the four Italian macro-regions.

**Table 3:** Number of overweight (a) and obese (b) people in Italy by gender and macro-regions, based on ISTAT (2019).

Age class	North-West		North-East		Centre		South-Islands	
	Male	Female	Male	Female	Male	Female	Male	Female
**(a) Overweight**								
18–24	107,602	48,923	81,693	37,340	95,845	43,268	183,415	84,754
25–34	257,920	125,603	192,241	94,677	236,117	115,720	432,168	210,912
35–44	407,112	222,414	305,082	167,831	371,976	208,786	582,341	324,154
45–54	591,508	298,292	445,693	225,227	517,866	273,641	762,243	406,452
55–64	490,347	358,948	372,306	271,455	442,268	334,247	685,246	517,492
65–74	419,781	360,664	309,840	264,159	372,895	323,797	568,786	484,237
75 or over	363,636	415,217	270,128	301,871	322,268	358,690	432,788	473,618
Total	2,637,906	1,830,061	1,976,982	1,362,559	2,359,234	1,658,150	3,646,987	2,501,619
%	40.4%	26.1%	41.8%	26.9%	42.7%	27.5%	47.9%	30.6%
**(b) Obese**								
18–24	13,565	10,004	11,657	8642	11,418	8361	22,394	16,785
25–34	50,221	37,922	42,366	32,352	43,444	33,014	81,498	61,671
35–44	107,643	70,161	91,298	59,921	92,936	62,235	149,122	99,033
45–54	158,666	108,211	135,311	92,474	131,262	93,801	198,020	142,800
55–64	157,662	132,715	135,487	113,595	134,372	116,776	213,385	185,304
65–74	122,792	128,741	102,579	106,722	103,070	109,216	161,135	167,404
75 or over	98,633	151,492	82,927	124,655	82,598	123,661	113,690	167,353
Total	709,184	639,245	601,625	538,361	599,101	547,063	939,245	840,350
%	10.9%	9.1%	12.7%	10.6%	10.8%	9.1%	12.3%	10.3%

Data is expressed in number of persons.

**Table 4:** Average BMI values for the Italian population by weight condition, gender, and macro-regions.

Macro-region	North-West		North-East		Centre		South-Islands	
Gender	Male	Female	Male	Female	Male	Female	Male	Female
Normal weight	22.34	21.68	22.36	21.72	22.42	21.75	22.64	21.93
Overweight	27.21	27.18	27.25	27.22	27.21	27.18	27.26	27.21
Obese	31.90	32.47	32.02	32.63	31.85	32.36	31.79	32.32

BMI is the ratio between weight (kg) and square of height (m). A BMI ranging from 18.5 to 25 is associated with a normal weight, values between 25 and 30 indicate an overweight condition, greater than 30 they indicate obesity.

The results are underestimated because data refers only to people over 18 years old. It means that the caloric intake in excess for overweight and obese younger people, which are a significant share of the total^14^, was not included in the evaluation.

### Definition of the standard diet

Data about daily food consumption was acquired from the third Italian National Food Consumption Survey^37^ which is the most updated available study on the topic to date, conducted from October 2005 to December 2006 and well documented by the National Research Institute for Food and Nutrition (INRAN). The INRAN database was organized in 15 large food categories and fifty-one subcategories, largely based on the classification developed by the European Food Safety Authority. In the present study, the original INRAN database was partially used for further analyses. In detail, the food consumption data were extracted only for the adult age group (18–64 years) and only 11 of 15 large categories, corresponding to 46 subcategories of foods, were considered. The daily consumption of potatoes was aggregated to vegetables, while meal substitutes; water and other non-alcoholic beverages; and miscellaneous categories were totally excluded because they were too generic to be considered as part of an idealized Italian diet. The daily food consumption is expressed in grams of product.

### Conversion of calories consumed in excess into quantities of food

Each food category included in the typical diet of the four Italian macro-regions is associated with a caloric content per 100 g. The specific caloric content that is considered for each of the food categories included in the typical diet, based on the corresponding INRAN data entry^38^ is reported in the Supplementary Information. This data is used to calculate the caloric content of the whole diet per day, with reference to the four Italian macro-regions (Table 1). The conversion rates between the kcal consumed in excess by overweight and obese people and the correspondent quantities of food are used to calculate the quantity of each food type corresponding to the kcal over-consumed by the population of each macro-region. Such quantities are then summed up to deliver the total quantity of food over-consumed by people in overweight and obese conditions.

### Environmental impact of overnutrition

The aim of the study is to analyse the environmental impact associated to FW due to overnutrition in Italy by applying the LCA methodology. According to the international standards of the ISO 14040 series^39^, LCA is an environmental methodology that allows us to estimate the impacts of production processes, through the quantification of inventory data from direct and indirect flows.

The functional unit of the study is the consumption of food by overweight and obese people in Italy over one year. Each foodstuff is modelled considering the cradle to consumer approach and including the following stages: farm or fishery, transport to the processing industry, industrial processing, packaging, transport from processing place to distribution centres and from distribution centres to retailer, storage at retailer, preparation at consumer and disposal of packaging. Transport from retailer to consumer and FW in the consumer’s household is excluded from the analysis. For each food item or group of foods, the inventory data are collected from the recently released Agribalyse 3.0 database. Agribalyse is the French Life Cycle Inventory database for the agriculture and food sector and the last version, published in 2020, contains LCIs for 2500 agricultural and food products.

The following assumptions and simplifications are made in this study:

- The impacts of each food category are obtained as the average of the impacts of the food items belonging to it without dressing or seasoning (e.g., for the rice food category, the following food items were considered: rice, cooked, unsalted; rice, brown, cooked, unsalted; rice, red, cooked, unsalted; wild rice, cooked, unsalted).
- LCI of each food product are modelled per 1 kg of prepared product, considering the mass-changing factors due to the inedible losses at consumption phase and the raw-to-cooked ratios; at this regard, data about the FW due to overnutrition in Italy, shown in Fig. 3, are assumed as the amount of cooked and edible food.
- In the food category “other meats”, two types of meat (rabbit and lamb) are considered.
- Agribalyse dataset is adapted to better represent Italian conditions in terms of the electricity mix.
- In line with the assumptions of the Agribalyse dataset, the secondary and tertiary packaging and the water use for the washing of fruits and vegetables are not accounted for.
- Some of the food items consumed in Italy are mainly imported from other countries, where different production systems may be adopted; the main limitation of this study is the assumption that the countries of origin of each raw material are the same as they are considered for food consumed in France.
- Changes in biomass and soil carbon stocks are not considered.

The environmental impact of overnutrition is estimated by calculating GHG emissions using the IPCC 2013 method^40^, multiplying the emission factors by the activity data. Simapro software has been used to model different foodstuffs with the Ecoinvent 3.2 database^41^ as a source of the background process for the Italian electricity mix.

## Supplementary Information

Supplementary Tables.

## References

[1] Toti E, Di Mattia C, Serafini M (2019). Metabolic food waste and ecological impact of obesity in FAO world’s region. Front. Nutr..

[2] Aschemann-Witzel J (2016). Waste not, want not, emit less. Science.

[3] Papargyropoulou E, Lozano R, Steinberger JK, Wright N, BinUjang Z (2014). The food waste hierarchy as a framework for the management of food surplus and food waste. J. Clean. Prod..

[4] Friedrich MJ (2017). Global obesity epidemic worsening. JAMA.

[5] Lobstein T, Cooper K (2020). Obesity: A ghost at the feast of the sustainable development goals. Curr. Obes. Rep..

[6] United Nations Environment Programme (UNEP) (2021). Food Waste Index Report 2021.

[7] Crippa M, Solazzo E, Guizzardi D, Monforti-Ferrario F, Tubiello FN, Leip A (2021). Food systems are responsible for a third of global anthropogenic GHG emissions. Nat. Food.

[8] Cattaneo A, Federighi G, Vaz S (2021). The environmental impact of reducing food loss and waste: A critical assessment. Food Policy.

[9] Serafini M, Toti E (2016). Unsustainability of obesity: Metabolic food waste. Front. Nutr..

[10] Barilla Centre for Food and Nutrition (BCFN) (2012). Food Waste: Causes, Impacts and Proposals.

[11] Smil V (2004). Improving efficiency and reducing waste in our food system. Environ. Sci..

[12] Ponis ST, Papanikolaou PA, Katimertzoglou P, Ntalla AC, Xenos KI (2017). Household food waste in Greece: A questionnaire survey. J. Clean. Prod..

[13] ISTAT (2019). Health Statistics—Body Mass Index, I.Stat Data Warehouse.

[14] Wang Y, Lobstein TIM (2006). Worldwide trends in childhood overweight and obesity. Int. J. Pediatr. Obes..

[15] Lauro R, Sbraccia P (2019). 1st Italian Obesity Barometer Report. Obesity Monitor.

[16] Edwards P, Roberts I (2008). Transport policy is food policy. The Lancet.

[17] Chaboud G, Daviron B (2017). Food losses and waste: Navigating the inconsistencies. Glob. Food Sec..

[18] Lettenmeier, M., Göbel, C., Liedtke, C., Rohn, H. & Teitscheid, P. Material Footprint of a sustainable nutrition system in 2050—Need for dynamic innovations in production, consumption and politics. In *Proc. Food System Dynamics*, 584–598 (2012).

[19] Società Italiana di Nutrizione Umana (SINU) (2014). LARN—Livelli di Assunzione di Riferimento di Nutrienti ed Energia per la Popolazione Italiana—IV Revisione.

[20] Battle-Bayer L, Bala A, García-Herrero I, Lemaire E, Song G, Aldaco R, Fullana-i-Palmer P (2019). The Spanish dietary guidelines: A potential tool to reduce greenhouse gas emissions of current dietary patterns. J. Clean. Prod..

[21] FAO (2021). Food and Agriculture Organization of the United Nations (FAO).

[22] ISMEA (2020). Gli scambi con l’estero dell’agroalimentare per anno.

[23] Buratti C (2017). Carbon footprint of conventional and organic beef production systems: An Italian case study. Sci. Total Environ..

[24] Bieńkowski J, Holka M, Dąbrowicz R, Jankowiak J (2018). Carbon footprint of beef cattle in a conventional production system: A case study of a large-area farming enterprise in the wielkopolska region. Probl. World Agric..

[25] Giordano C, Alboni F, Falasconi L (2019). Quantities, determinants, and awareness of households’ food waste in Italy: A comparison between diary and questionnaires quantities. Sustainability.

[26] ISPRA (2021). Italian Greenhouse Gas Inventory 1990-2019—National Inventory Report 2021. Rapporti 318/2020.

[27] Magkos F (2020). The environmental foodprint of obesity. Obesity.

[28] World Health Organization (WHO) (2016). Obesity and Overweight (Factsheet).

[29] Bender WH (1994). An end use analysis of global food requirements. Food Policy.

[30] FAO/WHO/UNU (2004). Human Energy Requirements.

[31] Hiç C, Pradhan P, Rybski D, Kropp JP (2016). Food surplus and its climate burdens. Environ. Sci. Technol..

[32] Schofield WN (1984). Predicting basal metabolic rate, new standards and review of previous work. Human nutrition. Clin. Nutr..

[33] Harris JA, Benedict FG (1918). A biometric study of human basal metabolism. Proc. Natl. Acad. Sci..

[34] Garrel DR, Jobin N, De Jonge LH (1996). Should we still use the Harris and Benedict equations?. Nutr. Clin. Pract..

[35] NCD-RisC. http://www.ncdrisc.org/data-downloads.html (2017). Accessed 14 Apr 2022.

[36] Franco S, Cicatiello C (2018). Food waste due to over-nutrition in the Italians’ dietary habits. Riv. Studi sulla Sostenibilita.

[37] INRAN-SCAI 2005–2006. www.crea.gov.it. Accessed 14 Apr 2022.

[38] INRAN. *Tabelle di composizione degli alimenti*. https://www.alimentinutrizione.it/sezioni/tabelle-nutrizionali (2019). Accessed 14 Apr 2022.

[39] ISO (2006). ISO 14040: 2006 Environmental Management—Life Cycle Assessment—Principles and Framework.

[40] Intergovernmental Panel on Climate Change (2014). Climate Change 2013: The Physical Science Basis. Working Group I Contribution to the Fifth Assessment Report of the Intergovernmental Panel on Climate Change.

[41] Steubing B, Wernet G, Reinhard J, Bauer C, Moreno-Ruiz E (2016). The Ecoinvent database version 3 (part II): Analyzing LCA results and comparison to version 2. Int. J. Life Cycle Assess..

