# Supplementary material for: Overnutrition is a significant component of food waste and has a large environmental impact
Source: Sci Rep. 2022 May 17;12:8166. doi: 10.1038/s41598-022-11813-5 (PMC9114125; doi:10.1038/s41598-022-11813-5)
Supplement: Supplementary file 1 — Supplementary Tables. [file 41598_2022_11813_MOESM1_ESM.docx]

**SUPPLEMENTARY INFORMATION**

Table 1. GHG emissions for each food category included in the Italian diet, calculated by using Agribalyse database (data are expressed in kg CO2-eq per kg of food). GHG emissions are calculated for each stage of the life cycle, the total appears in the right-end column.

| **Food category** | **Production** | **Transformation** | **Packaging** | **Transport** | **Distribution** | **Consumption** | **Total** |
| --- | --- | --- | --- | --- | --- | --- | --- |
| Beef meat | 43.09 | 0.11 | 0.39 | 0.27 | 0.04 | 0.14 | 44.02 |
| Beer | 0.04 | 0.25 | 0.50 | 0.27 | 0.03 | 0.00 | 1.09 |
| Biscuits | 4.33 | 0.35 | 0.11 | 0.15 | 0.03 | 0.00 | 4.97 |
| Bread | 0.27 | 0.16 | 0.10 | 0.13 | 0.01 | 0.00 | 0.66 |
| Breakfast cereals | 2.50 | 0.39 | 0.24 | 0.26 | 0.03 | 0.00 | 3.42 |
| Butter | 14.49 | 0.58 | 0.21 | 0.18 | 0.03 | 0.00 | 15.49 |
| Cheese | 5.23 | 0.27 | 0.26 | 0.26 | 0.03 | 0.00 | 6.06 |
| Chocolate | 16.46 | 1.32 | 0.10 | 0.37 | 0.01 | 0.00 | 18.28 |
| Citrus fruits | 0.20 | 0.00 | 0.00 | 0.28 | 0.04 | 0.22 | 0.74 |
| Cocoa | 25.68 | 0.28 | 0.40 | 0.40 | 0.01 | 0.00 | 26.78 |
| Crackers | 1.02 | 0.34 | 0.29 | 0.19 | 0.03 | 0.00 | 1.87 |
| Dessert | 2.80 | 0.44 | 0.21 | 0.18 | 0.03 | 0.00 | 3.66 |
| Dried fruit | 2.54 | 0.02 | 0.44 | 0.30 | 0.05 | 0.13 | 3.49 |
| Eggs | 2.45 | 0.05 | 0.13 | 0.25 | 0.05 | 0.28 | 3.21 |
| Fresh fish | 7.36 | 0.00 | 0.41 | 1.05 | 0.05 | 0.30 | 9.18 |
| Fresh leafy vegetables | 0.14 | 0.01 | 0.41 | 0.32 | 0.13 | 0.08 | 1.08 |
| Fresh tomatoes | 0.67 | 0.00 | 0.00 | 0.19 | 0.04 | 0.10 | 1.00 |
| Ham | 6.53 | 0.95 | 0.11 | 0.21 | 0.03 | 0.00 | 7.83 |
| Ice-creams | 1.44 | 0.29 | 0.21 | 0.20 | 0.05 | 0.02 | 2.20 |
| Legumes | 0.13 | 0.04 | 0.18 | 0.17 | 0.03 | 0.07 | 0.62 |
| Liqueurs | 0.09 | 0.31 | 0.52 | 0.20 | 0.01 | 0.00 | 1.14 |
| Marmalade | 0.42 | 0.25 | 0.55 | 0.35 | 0.03 | 0.00 | 1.61 |
| Milk | 1.19 | 0.01 | 0.17 | 0.10 | 0.01 | 0.00 | 1.49 |
| Offal | 30.86 | 0.07 | 0.40 | 0.27 | 0.04 | 0.07 | 31.70 |
| Olive oil | 1.13 | 0.28 | 0.22 | 0.15 | 0.03 | 0.00 | 1.80 |
| Other fats | 1.11 | 0.10 | 0.21 | 0.25 | 0.03 | 0.00 | 1.70 |
| Other fresh fruit-bearing vegetables | 1.24 | 0.00 | 0.00 | 0.20 | 0.05 | 0.07 | 1.57 |
| Other fresh vegetables | 0.46 | 0.00 | 0.00 | 0.21 | 0.06 | 0.20 | 0.91 |
| Other fruits | 0.25 | 0.00 | 0.03 | 0.18 | 0.04 | 0.09 | 0.60 |
| Other meats | 50.15 | 0.29 | 0.44 | 0.31 | 0.05 | 0.17 | 51.31 |
| Other oils | 1.54 | 0.12 | 0.22 | 0.13 | 0.03 | 0.00 | 2.03 |
| Other processed vegetables | 0.40 | 0.12 | 0.30 | 0.23 | 0.08 | 0.11 | 1.25 |
| Pasta | 2.55 | 0.33 | 0.24 | 0.22 | 0.03 | 0.01 | 3.40 |
| Pizza | 1.88 | 0.24 | 0.13 | 0.26 | 0.03 | 0.07 | 2.61 |
| Pork meat | 9.50 | 1.11 | 0.61 | 0.27 | 0.04 | 0.08 | 11.61 |
| Poultry meat | 4.87 | 1.45 | 0.44 | 0.35 | 0.05 | 0.15 | 7.31 |
| Preserved fish | 2.41 | 0.70 | 0.76 | 0.81 | 0.02 | 0.07 | 4.78 |
| Rice | 0.81 | 0.04 | 0.13 | 0.17 | 0.01 | 0.11 | 1.28 |
| Root vegetables | 0.14 | 0.01 | 0.41 | 0.32 | 0.13 | 0.08 | 1.08 |
| Spices | 0.26 | 0.00 | 0.39 | 0.18 | 0.05 | 0.05 | 0.93 |
| Sugar | 0.63 | 0.08 | 0.13 | 0.12 | 0.01 | 0.00 | 0.97 |
| Sweetness | 2.95 | 0.29 | 0.30 | 0.21 | 0.03 | 0.00 | 3.78 |
| Tropical fruits | 0.44 | 0.00 | 0.00 | 0.50 | 0.07 | 0.50 | 1.51 |
| Wheat flour | 0.35 | 0.01 | 0.00 | 0.12 | 0.02 | 0.00 | 0.51 |
| Wine | 0.25 | 0.06 | 0.54 | 0.25 | 0.02 | 0.00 | 1.13 |
| Yogurt | 1.66 | 0.15 | 0.21 | 0.19 | 0.03 | 0.00 | 2.24 |

Table 2. Calculated ER between overweight/obese and normal-weight people, by gender and age class, in the North-West (a), North-East (b), Centre (c) and South-Islands (d) of Italy and estimation of overnutrition per day by overweight and obese people. This data allows to calculate the kcal consumed in excess by overweight/obese persons, reported in the right end of the column.

| **a) NORTH-WEST** |  |  | **Weight (w)** | | | **Average BMR (kcal/day)** | | |  | **Average ER (kcal/day)** | | | **Overnutrition (kcal/day)** | |
| --- | --- | --- | --- | --- | --- | --- | --- | --- | --- | --- | --- | --- | --- | --- |
| **Male** | **Age (y)** | **Height (h)** | **Normal** | **Overweight** | **Obese** | **Normal** | **Overweight** | **Obese** | **PAL** | **Normal** | **Overweight** | **Obese** | **Overweight** | **Obese** |
| 18-24 | 21.0 | 178.0 | 70.8 | 86.2 | 101.1 | 1,788 | 2,001 | 2,205 | 1.70 | 3,040 | 3,401 | 3,749 | 361 | 709 |
| 25-34 | 29.6 | 176.9 | 69.9 | 85.2 | 99.8 | 1,713 | 1,923 | 2,125 | 1.65 | 2,826 | 3,172 | 3,506 | 346 | 679 |
| 35-44 | 39.8 | 175.6 | 68.9 | 83.9 | 98.4 | 1,624 | 1,830 | 2,029 | 1.60 | 2,598 | 2,928 | 3,247 | 331 | 649 |
| 45-54 | 49.5 | 174.4 | 67.9 | 82.7 | 97.0 | 1,539 | 1,743 | 1,939 | 1.55 | 2,385 | 2,701 | 3,005 | 316 | 620 |
| 55-64 | 59.3 | 173.2 | 67.0 | 81.6 | 95.6 | 1,453 | 1,654 | 1,848 | 1.50 | 2,180 | 2,481 | 2,771 | 301 | 592 |
| 65-74 | 69.3 | 171.9 | 66.0 | 80.4 | 94.3 | 1,366 | 1,564 | 1,754 | 1.40 | 1,912 | 2,189 | 2,456 | 277 | 544 |
| 75 or over | 81.4 | 170.4 | 64.8 | 79.0 | 92.6 | 1,260 | 1,455 | 1,642 | 1.30 | 1,638 | 1,891 | 2,135 | 253 | 496 |
| **Female** |  |  |  |  |  |  |  |  |  |  |  |  |  |  |
| 18-24 | 21.0 | 164.0 | 58.3 | 73.1 | 87.3 | 1,418 | 1,559 | 1,695 | 1.75 | 2,481 | 2,729 | 2,967 | 248 | 486 |
| 25-34 | 29.6 | 163.0 | 57.6 | 72.2 | 86.3 | 1,369 | 1,509 | 1,643 | 1.70 | 2,328 | 2,565 | 2,794 | 238 | 466 |
| 35-44 | 39.8 | 161.9 | 56.8 | 71.2 | 85.1 | 1,311 | 1,449 | 1,582 | 1.65 | 2,164 | 2,391 | 2,610 | 227 | 446 |
| 45-54 | 49.5 | 160.8 | 56.0 | 70.2 | 83.9 | 1,257 | 1,393 | 1,523 | 1.60 | 2,011 | 2,228 | 2,437 | 218 | 427 |
| 55-64 | 59.3 | 159.6 | 55.2 | 69.3 | 82.7 | 1,201 | 1,335 | 1,464 | 1.55 | 1,862 | 2,070 | 2,270 | 208 | 408 |
| 65-74 | 69.4 | 158.5 | 54.4 | 68.3 | 81.5 | 1,144 | 1,277 | 1,404 | 1.45 | 1,659 | 1,851 | 2,035 | 192 | 376 |
| 75 or over | 82.6 | 157.0 | 53.4 | 67.0 | 80.0 | 1,070 | 1,200 | 1,324 | 1.35 | 1,445 | 1,620 | 1,788 | 175 | 343 |
| **b) North-East** |  |  |  |  |  |  |  |  |  |  |  |  |  |  |
| **Male** |  |  |  |  |  |  |  |  |  |  |  |  |  |  |
| 18-24 | 21.0 | 180.0 | 72.4 | 88.3 | 103.7 | 1,821 | 2,040 | 2,252 | 1.70 | 3,096 | 3,467 | 3,828 | 371 | 732 |
| 25-34 | 29.6 | 178.9 | 71.6 | 87.2 | 102.5 | 1,746 | 1,961 | 2,171 | 1.65 | 2,881 | 3,236 | 3,582 | 356 | 702 |
| 35-44 | 39.8 | 177.6 | 70.5 | 86.0 | 101.0 | 1,656 | 1,869 | 2,075 | 1.60 | 2,650 | 2,990 | 3,321 | 340 | 671 |
| 45-54 | 49.5 | 176.4 | 69.6 | 84.8 | 99.6 | 1,571 | 1,781 | 1,985 | 1.55 | 2,435 | 2,760 | 3,076 | 325 | 641 |
| 55-64 | 59.3 | 175.2 | 68.6 | 83.6 | 98.2 | 1,485 | 1,692 | 1,893 | 1.50 | 2,228 | 2,538 | 2,840 | 310 | 611 |
| 65-74 | 69.3 | 173.9 | 67.6 | 82.4 | 96.8 | 1,398 | 1,602 | 1,800 | 1.40 | 1,957 | 2,242 | 2,520 | 285 | 562 |
| 75 or over | 81.4 | 172.4 | 66.4 | 81.0 | 95.1 | 1,292 | 1,492 | 1,687 | 1.30 | 1,680 | 1,940 | 2,193 | 260 | 513 |
| **Female** |  |  |  |  |  |  |  |  |  |  |  |  |  |  |
| 18-24 | 21.0 | 166.0 | 59.8 | 75.0 | 89.9 | 1,436 | 1,581 | 1,724 | 1.75 | 2,514 | 2,767 | 3,017 | 254 | 503 |
| 25-34 | 29.6 | 165.0 | 59.1 | 74.1 | 88.8 | 1,388 | 1,531 | 1,672 | 1.70 | 2,359 | 2,602 | 2,842 | 244 | 483 |
| 35-44 | 39.8 | 163.9 | 58.3 | 73.1 | 87.6 | 1,330 | 1,471 | 1,610 | 1.65 | 2,194 | 2,427 | 2,656 | 233 | 462 |
| 45-54 | 49.5 | 162.8 | 57.5 | 72.1 | 86.4 | 1,275 | 1,414 | 1,551 | 1.60 | 2,040 | 2,263 | 2,482 | 223 | 442 |
| 55-64 | 59.3 | 161.6 | 56.7 | 71.1 | 85.2 | 1,219 | 1,357 | 1,492 | 1.55 | 1,890 | 2,103 | 2,313 | 213 | 422 |
| 65-74 | 69.4 | 160.5 | 55.9 | 70.1 | 84.0 | 1,162 | 1,298 | 1,431 | 1.45 | 1,685 | 1,882 | 2,075 | 196 | 390 |
| 75 or over | 82.6 | 159.0 | 54.9 | 68.8 | 82.5 | 1,088 | 1,221 | 1,352 | 1.35 | 1,469 | 1,648 | 1,825 | 179 | 356 |

| **c) Centre** |  |  |  |  |  |  |  |  |  |  |  |  |  |  |
| --- | --- | --- | --- | --- | --- | --- | --- | --- | --- | --- | --- | --- | --- | --- |
| **Male** |  |  |  |  |  |  |  |  |  |  |  |  |  |  |
| 18-24 | 21.0 | 178.0 | 71.0 | 86.2 | 100.9 | 1,792 | 2,001 | 2,203 | 1.70 | 3,046 | 3,402 | 3,745 | 355 | 699 |
| 25-34 | 29.6 | 176.9 | 70.2 | 85.2 | 99.7 | 1,717 | 1,923 | 2,122 | 1.65 | 2,832 | 3,173 | 3,502 | 340 | 670 |
| 35-44 | 39.8 | 175.6 | 69.1 | 83.9 | 98.2 | 1,627 | 1,831 | 2,027 | 1.60 | 2,603 | 2,929 | 3,243 | 325 | 640 |
| 45-54 | 49.5 | 174.4 | 68.2 | 82.8 | 96.9 | 1,542 | 1,743 | 1,937 | 1.55 | 2,391 | 2,701 | 3,002 | 311 | 611 |
| 55-64 | 59.3 | 173.2 | 67.2 | 81.6 | 95.5 | 1,457 | 1,654 | 1,845 | 1.50 | 2,185 | 2,481 | 2,768 | 297 | 583 |
| 65-74 | 69.3 | 171.9 | 66.2 | 80.4 | 94.1 | 1,369 | 1,564 | 1,752 | 1.40 | 1,917 | 2,190 | 2,453 | 273 | 536 |
| 75 or over | 81.4 | 170.4 | 65.1 | 79.0 | 92.4 | 1,264 | 1,455 | 1,640 | 1.30 | 1,643 | 1,891 | 2,132 | 249 | 489 |
| **Female** |  |  |  |  |  |  |  |  |  |  |  |  |  |  |
| 18-24 | 21.0 | 164.0 | 58.5 | 73.1 | 87.0 | 1,420 | 1,559 | 1,693 | 1.75 | 2,484 | 2,729 | 2,962 | 244 | 478 |
| 25-34 | 29.6 | 163.0 | 57.8 | 72.2 | 86.0 | 1,371 | 1,509 | 1,641 | 1.70 | 2,331 | 2,565 | 2,789 | 235 | 459 |
| 35-44 | 39.8 | 161.9 | 57.0 | 71.2 | 84.8 | 1,313 | 1,449 | 1,579 | 1.65 | 2,167 | 2,391 | 2,606 | 224 | 439 |
| 45-54 | 49.5 | 160.8 | 56.2 | 70.2 | 83.6 | 1,258 | 1,393 | 1,521 | 1.60 | 2,013 | 2,228 | 2,433 | 215 | 420 |
| 55-64 | 59.3 | 159.6 | 55.4 | 69.3 | 82.5 | 1,203 | 1,335 | 1,462 | 1.55 | 1,865 | 2,070 | 2,266 | 205 | 401 |
| 65-74 | 69.4 | 158.5 | 54.6 | 68.3 | 81.3 | 1,146 | 1,277 | 1,401 | 1.45 | 1,662 | 1,851 | 2,032 | 189 | 370 |
| 75 or over | 82.6 | 157.0 | 53.6 | 67.0 | 79.8 | 1,072 | 1,200 | 1,322 | 1.35 | 1,447 | 1,620 | 1,785 | 173 | 338 |
| **d) South-Islands** |  |  |  |  |  |  |  |  |  |  |  |  |  |  |
| **Male** |  |  |  |  |  |  |  |  |  |  |  |  |  |  |
| 18-24 | 21.0 | 176.0 | 70.1 | 84.4 | 98.5 | 1,770 | 1,966 | 2,160 | 1.70 | 3,008 | 3,343 | 3,671 | 335 | 663 |
| 25-34 | 29.6 | 174.9 | 69.3 | 83.4 | 97.3 | 1,694 | 1,889 | 2,079 | 1.65 | 2,795 | 3,116 | 3,431 | 321 | 636 |
| 35-44 | 39.8 | 173.6 | 68.2 | 82.2 | 95.8 | 1,605 | 1,796 | 1,984 | 1.60 | 2,568 | 2,874 | 3,175 | 307 | 607 |
| 45-54 | 49.5 | 172.4 | 67.3 | 81.0 | 94.5 | 1,520 | 1,709 | 1,894 | 1.55 | 2,356 | 2,648 | 2,936 | 293 | 580 |
| 55-64 | 59.3 | 171.2 | 66.3 | 79.9 | 93.1 | 1,434 | 1,620 | 1,803 | 1.50 | 2,151 | 2,430 | 2,704 | 279 | 553 |
| 65-74 | 69.3 | 169.9 | 65.3 | 78.7 | 91.8 | 1,347 | 1,530 | 1,710 | 1.40 | 1,886 | 2,142 | 2,394 | 257 | 509 |
| 75 or over | 81.4 | 168.4 | 64.2 | 77.3 | 90.1 | 1,241 | 1,421 | 1,598 | 1.30 | 1,614 | 1,848 | 2,078 | 234 | 464 |
| **Female** |  |  |  |  |  |  |  |  |  |  |  |  |  |  |
| 18-24 | 21.0 | 162.0 | 57.5 | 71.4 | 84.8 | 1,407 | 1,540 | 1,668 | 1.75 | 2,462 | 2,694 | 2,919 | 232 | 457 |
| 25-34 | 29.6 | 161.0 | 56.8 | 70.6 | 83.8 | 1,358 | 1,489 | 1,616 | 1.70 | 2,309 | 2,532 | 2,747 | 223 | 438 |
| 35-44 | 39.8 | 159.9 | 56.0 | 69.5 | 82.6 | 1,301 | 1,430 | 1,555 | 1.65 | 2,146 | 2,359 | 2,565 | 213 | 419 |
| 45-54 | 49.5 | 158.8 | 55.3 | 68.6 | 81.5 | 1,246 | 1,373 | 1,496 | 1.60 | 1,993 | 2,197 | 2,394 | 204 | 401 |
| 55-64 | 59.3 | 157.6 | 54.5 | 67.6 | 80.3 | 1,190 | 1,316 | 1,437 | 1.55 | 1,845 | 2,040 | 2,228 | 195 | 383 |
| 65-74 | 69.4 | 156.5 | 53.7 | 66.6 | 79.1 | 1,134 | 1,257 | 1,377 | 1.45 | 1,644 | 1,823 | 1,997 | 180 | 353 |
| 75 or over | 82.6 | 155.0 | 52.7 | 65.4 | 77.6 | 1,059 | 1,181 | 1,298 | 1.35 | 1,430 | 1,594 | 1,752 | 164 | 322 |

Table 3. Caloric content of the 46 food categories considered in the Italian typical diet. The caloric content is estimated on the base of the INRAN database (INRAN, 2019, reference n. [39]). For some food categories, the caloric content has been estimated as the average of different foods included in that category. Original data are available at <https://www.alimentinutrizione.it/sezioni/tabelle-nutrizionali>

| **Food category** | **Kcal/100g** | **INRAN code** |
| --- | --- | --- |
| Beef meat | 145 | 101120 |
| Beer | 34 | 404010 |
| Biscuits | 426 | 000900 |
| Bread | 268 | 000530 |
| Breakfast cereals | 354 | 003050 |
| Butter | 758 | 190010 |
| Cheese | 345 | Average between 160820 and 164820 and 166000 |
| Chocolate | 542 | Average between 203010 and 203020 |
| Citrus fruits | 37 | 008000 |
| Cocoa | 355 | 508010 |
| Crackers | 386 | 001020 |
| Dessert | 140 | 140100 |
| Dried fruit | 524 | Average between 008810 and 008540 and 008570 |
| Eggs | 128 | 181100 |
| Fresh fish | 118 | Average between 121490 and 123220 and 127100 and 127170 |
| Fresh leafy vegetables | 22 | 005410 |
| Fresh tomatoes | 19 | 006600 |
| Ham | 271 | 110520 |
| Ice-creams | 178 | Average between 206040 and 206710 |
| Legumes | 96 | Average between 004510 and 004010 and 004210 |
| Liqueurs | 314 | 407010 |
| Marmalade | 227 | 204500 |
| Milk | 64 | 135010 |
| Offal | 142 | 115320 |
| Olive oil | 899 | 009210 |
| Other fats | 760 | 009100 |
| Other fresh fruit-bearing vegetables | 22 | Average between 005600 and 005500 and 005730 |
| Other fresh vegetables | 22 | Same value as “Other fresh fruit-bearing vegetables” |
| Other fruits | 41 | Average between 007120 and 007290 and 007000 and 007570 |
| Other meats | 110 | Average between 107010 and 104030 |
| Other oils | 899 | 009650 |
| Other processed vegetables | 22 | 006680 |
| Pasta | 341 | 000800 |
| Pizza | 306 | 000700 |
| Pork meat | 157 | 105230 |
| Poultry meat | 100 | 106500 |
| Preserved fish | 103 | 123550 |
| Rice | 338 | 000120 |
| Root vegetables | 72 | 006500 |
| Spices | 30 | 006840 |
| Sugar | 392 | 201500 |
| Sweetness | 339 | 208510 |
| Tropical fruits | 76 | 007510 |
| Wheat flour | 323 | 000220 |
| Wine | 73 | Average between 405010 and 405020 |
| Yogurt | 66 | 150010 |
